# Determinants of early initiation of breastfeeding in Ethiopia: a systematic review and meta-analysis

**DOI:** 10.1186/s12887-026-07237-z

**Published:** 2026-07-02

**Authors:** Sahardiid Ali Abdilahi, Mohamed Omar Osman, Kalkidan Hassen Abate, Jemal Haidar Ali

**Affiliations:** 1 Departments of Public Health, College of Medicine and Health Sciences, Jigjiga University, P.O.BOX: 1020, Jigjiga, Ethiopia; 2 Department of Population and Family Health, Jimma University, P.O. BOX: 378, Jimma, Ethiopia; 3 College of Health Science, School of Public Health, Addis Ababa University, P.O. Box 27285 1000 , Addis Ababa, Ethiopia; 4 Graduate School of Medicine, University of Wollongong, Wollongong, NSW 2522 Australia

**Keywords:** A systematic review, Breast feeding, Determinants, Early initiation, Ethiopia: meta-analysis

## Abstract

**Background:**

Timely initiation of breastfeeding is among the major public health problems that contribute to childhood undernutrition, morbidity, mortality, impaired intellectual development, suboptimal adult work capacity, and increased risk of non-communicable chronic diseases worldwide, including in Ethiopia Nevertheless, the country-specific determinants of early initiation of breastfeeding in Ethiopia show inconsistent results, and therefore we aimed at synthesizing the existing literature on early initiation of breastfeeding in Ethiopia. While previous national systematic reviews have aggregated broad institutional data, local findings remain highly divergent, and an updated synthesis filtering for rigorous adjustment of confounding factors is lacking. This study aims to bridge this evidence gap by pooling contemporary data to provide clearer policy relevance for maternal and child health interventions.

**Methods:**

Biomedical electronic databases, including PubMed/Medline, HINARI, Science Direct, African Journal Online (AJOL), and Google Scholar, based on the guidelines of the Preferred Reporting Items for Systematic Reviews and Meta-Analyses (PRISMA), were used, and the protocol was registered in PROSPERO (CRD420261307602). The available literature was critically reviewed, and STATA software version 13 was used to analyses the extracted data. Heterogeneity among studies was assessed using I2 and meta-bias statistics for publication bias. A random-effects model with a 95% confidence interval was used for pooled estimations.

**Results:**

Of the 146 articles screened, 19 of them revealed that mothers residing in urban areas OR = OR = 2.02 (95% CI: 1.31–3.10), counselled during antenatal care OR = OR = 2.97 (95% CI: 2.00–4.41), advised during postnatal care OR = 6.27 (95% CI: 2.09–18.74), had normal delivery (spontaneous vertex delivery) OR = 2.99 (95% CI: 1.27–7.06), delivered at health institutions OR = 5.24 (95% CI: 2.16–12.74), and did not practice pre-lacteal feeding to their newborns OR = 7.95 (95% CI: 3.62–17.47) were the significant factors associated with early initiation of breastfeeding in Ethiopia.

**Conclusion:**

This review provides consolidated evidence regarding the key structural, institutional, and behavioral predictors of EIBF in Ethiopia. Because the primary data are derived from observational designs, these associations reflect strong prognostic indicators rather than definitive causal links. To increase the practice of early breastfeeding initiation, a combination of actions like behavioral change communication and the need for expanded maternal healthcare access, addressing home birth barriers, as well as implementing health service protocols are recommended.

**Supplementary Information:**

The online version contains supplementary material available at https://doi.org/10.1186/s12887-026-07237-z.

## Introduction

The WHO defines early initiation of breastfeeding as putting newborns to the breast within the first hour of life after delivery, which is considered an indicator of infants’ health and makes sure they get their “first milk”, or colostrum, which is full of protecting elements. The World Health Organization and the United Nations Children’s Fund recommend early initiation of breastfeeding within 1 h of birth [1].

An estimated 2.7 million children die each year from undernutrition, accounting for 45% of all child deaths. Infant and young child feeding (IYCF) is a key area to improve child survival and promote their healthy growth and development [2]. During the first two years of a child’s life, adequate nutrition is crucial since it lowers the risk of morbidity and mortality as well as chronic diseases and promotes their overall health development [3].

Breastfeeding is among the key infant feeding practices worldwide and a highly effective public health intervention, providing long-term benefits including a reduction in obesity risk and type 2 diabetes and improved cognitive development, as well as short-term benefits including enhanced gut microbiome colonization, protection against infection and lower infant mortality [4, 5].

Breast milk is a biofluid that has been shown to include a wide range of macro- and micronutrients and is assumed to provide the nutritional needs of newborns. Its composition shows dynamic behavior, changing over time to adapt to the baby’s needs [6].

Early initiation of breastfeeding within the first hour of birth reduces significantly neonatal mortality and the risk of infections such as pneumonia, sepsis, and diarrhea while also promoting sustained breastfeeding and long-term child development [7].

Studies also found that early initiation of breastfeeding within the first hour of birth reduces neonatal mortality and infection; infants who begin breastfeeding later compared to those who begin early have about a 30% higher risk of neonatal death, and initiation supports sustained breastfeeding [8, 9].

Globally, an estimated 77 million newborns miss out on the lifesaving advantages of early breastfeeding, which emphasizes the urgency of early initiation of breastfeeding. Delayed breastfeeding initiation increases the risk of neonatal mortality nearly by 40% when started two to three hours after birth and by up to 80% when delayed into 24 h and beyond [10].

The 2010 and 2018 Demographic and Health Surveys (DHS) conducted worldwide revealed the weighted prevalence of early breastfeeding initiation (EIBF) of 51.9%, of which African countries accounted for 55.8%, while American, Eastern Mediterranean, European, and South-East Asian/Western Pacific countries constituted 61.2%, 42.6%, 67%, and 47.4%, respectively [11].

Based on data from a meta-analysis done across 29 sub-Saharan African countries, the prevalence of EIBF in infants aged 0–23 months was 46.94% in West Africa, 61.82% in East Africa, 37.84% in Central Africa, and 69.31% in Southern Africa, in which Guinea (16.54%), Kenya (30.38%), and Congo Brazzaville (24.10%) [12]. A multilevel study conducted in 32 Sub-Saharan African nations between 2010 and 2020 revealed the pooled prevalence of EIBF in these countries was 57%; of which, Burundi had the highest rate of EIBF (86%), while Congo Brazzaville had the lowest rate (24%) [13], and Lesotho (66.24%) was the nation with the highest prevalence of EIBF [12]. On the other hand, the prevalence of EIBF conducted in two city administrations and nine regional states of Ethiopia is also 75.2% [14].

Despite the enormous protective benefits of timely EIBF, the issue still remained as one of the major worldwide health challenges since its practice is low. Based on previous research reports, timely EIBF is documented to play an important role in reducing child morbidity and mortality globally [15].

Some previous studies conducted elsewhere have identified a range of factors that influence the timely EIBF. These factors included the wealth index, the place and mode of delivery, the woman’s educational status, media exposure, caesarean section, the baby’s birth weight, the mothers’ awareness of BF, newborn sex, maternal age, the presence of a professional birth attendant, maternal education, low birthweight, urban settings, postnatal counselling, and child characteristics such as birth order, male gender, micronutrient consumption, and immediate skin-to-skin contact [13, 16, 21].

In Ethiopia, however, the information on the determinants of EIBF documented are disparate and inconsistent results, suggesting the need for country-specific profiling of the determinants. With this background information, we conducted the study to uncover the driving factors of EIBF contextually that may offer new perspectives on how to improve and expand early initiation of breastfeeding initiatives in Ethiopia. With the increased global efforts to promote child nutrition and eliminate all types of malnutrition, Ethiopian policy decision-makers and public health practitioners would find this information helpful in offering targeted policy interventions.

Although prior broad systematic reviews on child health in Ethiopia exist, their conclusions are often limited by the simultaneous pooling of crude and adjusted estimates, introducing substantial confounding bias. Furthermore, older reviews integrated a larger number of primary studies without filtering out papers suffering from structural missingness or unstandardized definitions of maternal exposures. This current review provides a distinct scientific rationale and added value by implementing a strict quality-controlled pool of 19 highly homogeneous, adjusted-model observational studies. By synthesizing only multi-variable controlled data up to 2024, this review resolves conflicting regional reports and delivers highly precise, epidemiologically sound indicators to guide national newborn feeding policies in Ethiopia. Therefore, this study is aimed at finding pooled results on early initiation of breastfeeding (EIBF) in Ethiopia.

## Methods

### Protocol and registration

The protocol titled ‘Predictors of early initiation of breastfeeding in Ethiopia: a systematic review and meta-analysis’ was registered with the International Prospective Register of Systematic Reviews (PROSPERO) at the University of York Centre for Reviews and Dissemination (https://www.crd.york.ac.uk/) with the registration number (CRD420261307602).

### Search strategy

We used the biomedical electronic databases PubMed/Medline, HINARI, Science Direct, African Journal Online (AJOL), and Google Scholar for a thorough literature search. To find more literature, the reference lists of research that qualified were also looked through. All published articles from 2010 to 2024 were included in the search. This study was aimed at assessing predictors of early initiation of breastfeeding among children born in Ethiopia because previous studies failed to come up with results that can be used as policy and practice input. The Medical Subject Headings were searched for using either one or more of the following terms. (MeSH terms): Using keywords as the search terms, we included alternative terms for ‘determinant’ and combined them by using Boolean operators. Search terms. Search (determinants OR associated factors OR predictors) AND (early initiation of breastfeeding OR breastfed OR breastfeeding OR breastfed OR feeding OR breastfeeding OR wet nursing OR milk sharing OR sharing milk) AND Ethiopia. The Preferred Reporting Items for Systematic Reviews and Meta-Analyses Protocols (PRISMA-P) checklist guideline was followed throughout our investigation [22].

### Exposure

Maternal, institutional, clinical, and environmental predictors or exposures (such as ANC counseling, urban/rural residence, mode of delivery, and place of birth) hypothesized to influence infant feeding habits.

### Outcome

The primary outcome of interest is early initiation of breastfeeding (EIBF) among infants, strictly defined as the initiation of breastfeeding within the first hour of life.

### Measurement of the outcome variable

The aim of this review was to assess predictors of EIBF in Ethiopia.

### Inclusion and exclusion criteria

Studies were selected for the systematic reviews and meta-analysis if they were done in Ethiopia, written in English, and reported the determinants of and/or associated factors and/or predictors of EIBF. After the initial screening of all the titles obtained from our searches, all abstracts were then assessed for eligibility by two authors (SA and MO) independently, as formed by the inclusion criteria; disagreements between the two investigators were fixed through consensus and discussion. Finally, studies that met the following criteria were incorporated in the meta-analysis: all observational studies conducted on the issue in Ethiopia, featuring a response rate of 80% or higher, were included. This review did not include reviews, studies with systematically poor design, grey literature, such as case reports or interventional studies, articles without full text and data that are difficult to extract, or research papers published in languages other than English. A factor was considered for inclusion if it was found to be associated with early initiation of breastfeeding in two or more studies (articles). If the association was found in only one study, it was not considered for inclusion. Advice on breastfeeding (BF) at postnatal (PNC), birth attendants, counselling during antenatal care (ANC), and mode of delivery: Education status, knowledge about early life, place of birth, and residence were taken into consideration when analyzing the contributing factors to EIBF.

### Data extraction/abstraction

The data extraction was conducted by two independent researchers (SA and MO) using a standardized format for determinant studies. The data extraction was conducted by two independent researchers (SA and MO) using a standardized format for determinant studies. Crucially, only the adjusted odds ratios (AOR) and accompanying 95% confidence intervals were extracted for pooling to control for the effects of residual confounding variables present in individual primary studies.

### Operationalization of outcome measures

The main outcome of interest quantified across all primary studies was the proportion of neonates undergoing EIBF. To accurately evaluate the independent strength of the associations, the extracted multi-variable Adjusted Odds Ratios (AOR) were compiled into standard data extraction matrices. Crude association values (COR) were completely excluded from synthesis to protect the validity of the final pooled results.

### Synthesis of results/statistical analysis

Information about the studies was gathered using the two-by-two table method from various studies and using Microsoft Excel and then exported to STATA version 13 software (Stata-Corp. LP, College Station, TX, USA). Finally, STATA version 13 was used for data analysis. The software was chosen because of its extensive functionalities for combining data from several studies, which is a basic requirement for performing meta-analytical research. Using a forest plot, the odds ratio (OR) with 95% confidence interval (CI), P-value, and I^2^ were displayed. Heterogeneity among studies was assessed using an I² statistic with values ranging from 0% to 100%: low heterogeneity for < 50%, moderate heterogeneity for 50%-75%, and high heterogeneity for greater than or equal to 75% [23].

The first step in determining whether publication bias is present is to utilize a funnel plot, which is generally reserved for meta-analyses of 10 or more papers. Publication bias was assessed using the funnel plot’s visual appraisal for meta-analyses that included a minimum of ten papers [24], and then Eggers’ regression test was conducted to determine the presence of publication bias when the p-value < 0.10 [25].

### Quality assessment for studies

Following a quality evaluation, studies meeting the JBI’s nine criteria scoring five or higher were considered for inclusion in this review and meta-analysis [26], The exhaustive, study-level item breakdown scoring matrix for all 19 included papers is provided as Supplementary Tables 2 to ensure full methodological transparency. Additionally, to assess the overall certainty and strength of the evidence generated by these pooled observational studies, a formal GRADE (Grading of Recommendations Assessment, Development and Evaluation) certainty assessment was completed for each major predictor pathway, as detailed in the Results section [27].

## Results

**Fig. 1:**
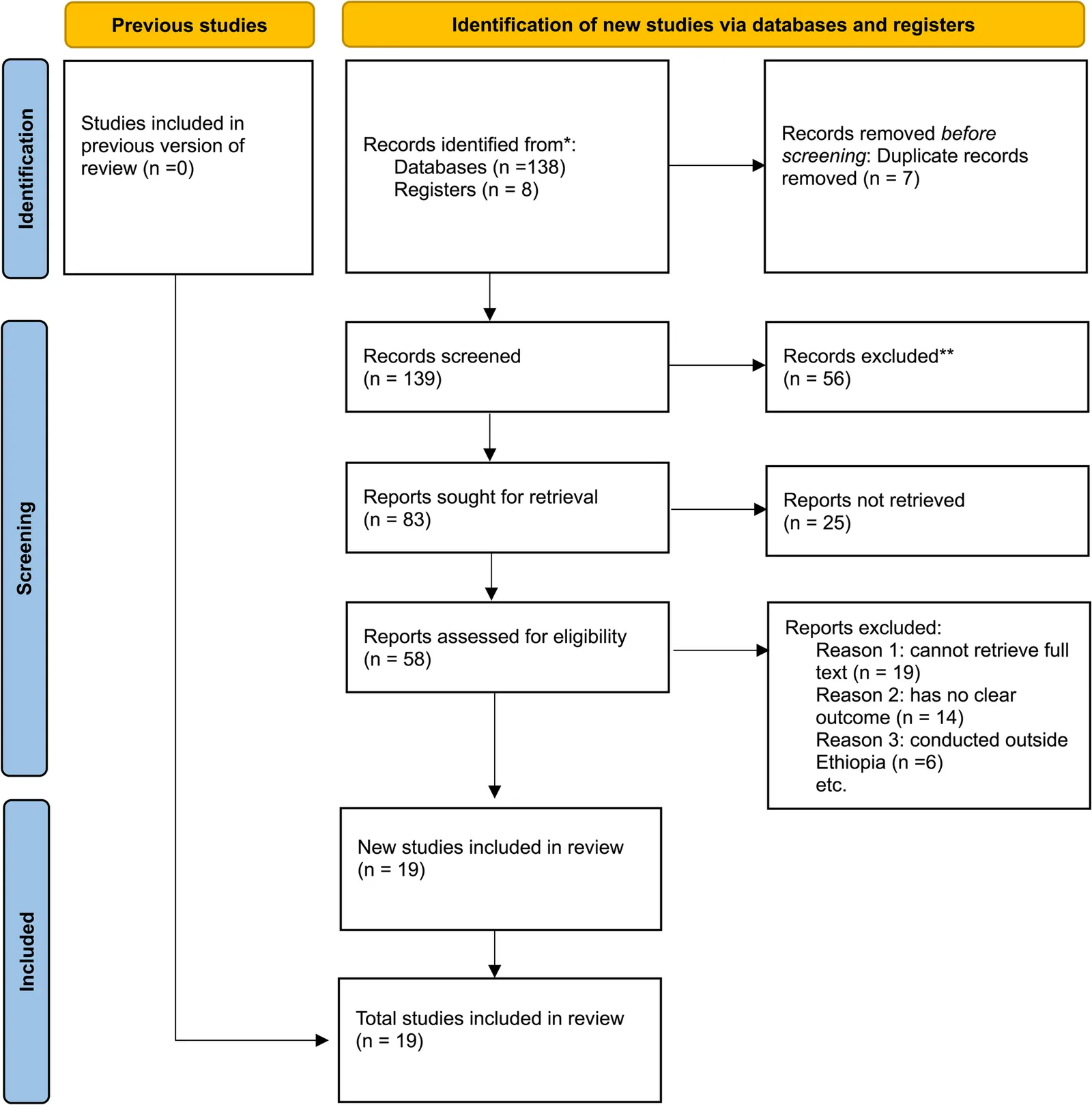
A flow diagram of the studies included in the review of determinants of early breastfeeding initiation in Ethiopia

Based on the search engines used (PubMed/Medline, Hinari, African Journal Online (AJOL), Google Scholar, Science Direct, and other electronic databases were used for the database search and desk review). The initial multi-database electronic search yielded a total of 146 records. Following the removal of 7 duplicate entries, 139 unique articles underwent strict title and abstract screening. Of these, 56 articles were excluded due to complete irrelevance to the primary EIBF outcome. This left 83 articles for full-text retrieval, of which 25 reports could not be successfully retrieved. The remaining 58 articles were subjected to exhaustive full-text eligibility appraisal against our predefined quality parameters. Ultimately, 39 articles were excluded with documented reasons: 19 lacked accessible complete texts, 14 displayed unclear or unextractable outcome metrics, and 6 were conducted completely outside of the geographic boundaries of Ethiopia. This rigorous filtering process resulted in a final stable sample of 19 unique, high-quality primary studies for complete meta-analysis synthesis (Fig. 1).

### Characteristics of the articles included

As shown in Table 1, nineteen studies were included, pooled from the relevant articles. Further analysis was done on these ten variables that determined EIBF in Ethiopia. The studies included both cross-sectional and longitudinal studies. A total of 17,529 mothers of children aged 0 to 24 months were included in the analysis. The included studies had sample sizes ranging from 182 [28] to 6,761 [29]. Factors such as counseling during antenatal care [28, 38], postnatal care advice [18, 28, 35, 39], knowledge about early-initiation breastfeeding [33, 40], practicing colostrum feeding [28, 35, 36], mode of delivery [31, 32, 34, 38, 40, 42], delivery attendants [42, 43], place of delivery [31, 33, 35, 38], pre-lacteal feeding [29, 35, 42], residence [18, 29, 44], and primary education and above [34, 39], were found to be included (Table 1).

**Table 1:** Characteristics of the included studies

S/*N*	Author	Region	Study design	Year	Population Characteristics	*N*	JBI score
1	(Setegn, Gerbaba et al. 2011) [18]	Oromia	Cross-sectional	2011	-Residence -Advise on breastfeeding at post-natal care	608	8
2	(Woldemichael and Kibie 2016) [39]	Oromia	Cross-sectional	2016	-Primary education and above -Place of delivery -Advise on breastfeeding at post-natal care	386	9
3	(Wolde, Birhanu et al. 2014) [28]	Oromia	Cross-sectional	2014	-Antenatal care follow up - Colostrum feeding	182	7
4	(Tilahun, Degu et al. 2016) [36]	Amhara	Cross-sectional	2016	-Antenatal care follow up - Mode of delivery - Delivery attendant - Colostrum feeding	409	8
5	(Ayalew, Kassie et al. 2022) [30]	Amhara	Cross-sectional	2022	- Counseling during antenatal care	569	9
6	(Belachew 2019) [32]	Amhara	Cross-sectional	2019	- Counseling during antenatal care -Mode of delivery -Place of delivery	472	8
7	(Yohannes, Tesfaye et al. 2020) [38]	SNNP	Cross-sectional	2020	- Counseling during antenatal care -Mode of delivery -Place of delivery	333	7
8	(Deressa, Tadese et al. 2017) [33]	Benishangul Gumuz	Cross-sectional	2017	-Place of delivery - Counseling during antenatal care Knowledge about of early initiation breast feeding	770	8
9	(Berhe, Mekonnen et al. 2013) [41]	Tigray	Cross-sectional	2013	-Place of delivery -Mode of delivery - Delivery attendant	361	6
10	(Mekonnen and Shewangizaw 2021) [42]	Addis Ababa	Cross-sectional	2022	-Pre lacteal feeding - Delivery attendant -Mode of delivery	322	6
11	(Gemeda, Chekole et al. 2022) [35]	Dire Dawa	Cross-sectional	2022	- Counseling during antenatal care -Place of delivery -Mode of delivery -Advise on breastfeeding at post-natal care -Pre lacteal feeding	302	6
12	(Derso, Biks et al. 2017) [29]	Amhara	Survey	2017	-Residence -Counseling during antenatal care -Pre lacteal feeding	6,761	9
13	(Gebremeskel, Gebru et al. 2019) [34]	Tigray	Cross-sectional	2019	-Primary education and above -Mode of delivery	803	8
14	(Deme, Bekele et al. 2015) [63]	Oromia	Cross-sectional	2015	-Place of delivery	422	7
15	(Gebretsadik, Tkuwab et al. 2020) [43]	Afar	Cross-sectional	2020	- Delivery attendant	390	6
16	(Ayalew, Tewabe et al. 2019) [31]	Amhara	Cross-sectional	2019	- Counseling during antenatal care -Place of delivery -Mode of delivery	423	8
17	(Armdie, Ejigu et al. 2024) [16]	Ethiopia	Longitudinal Survey	2024	-Residence -Mode of delivery -Delivery attendant -Advise on breastfeeding at post-natal care	2564	7
18	(Worku, Tilahun; et al. 2014) [37]	Oromia	Cross-sectional	2014	- Counseling during antenatal care -Mode of delivery	633	6
19	(Musa Seid 2014) [40]	Amhara	Cross-sectional	2014	-Mode of delivery -Knowledge about of early initiation breast feeding	819	8

### Determinant factors associated with the early initiation of breast feeding in Ethiopia

**Fig. 2:**
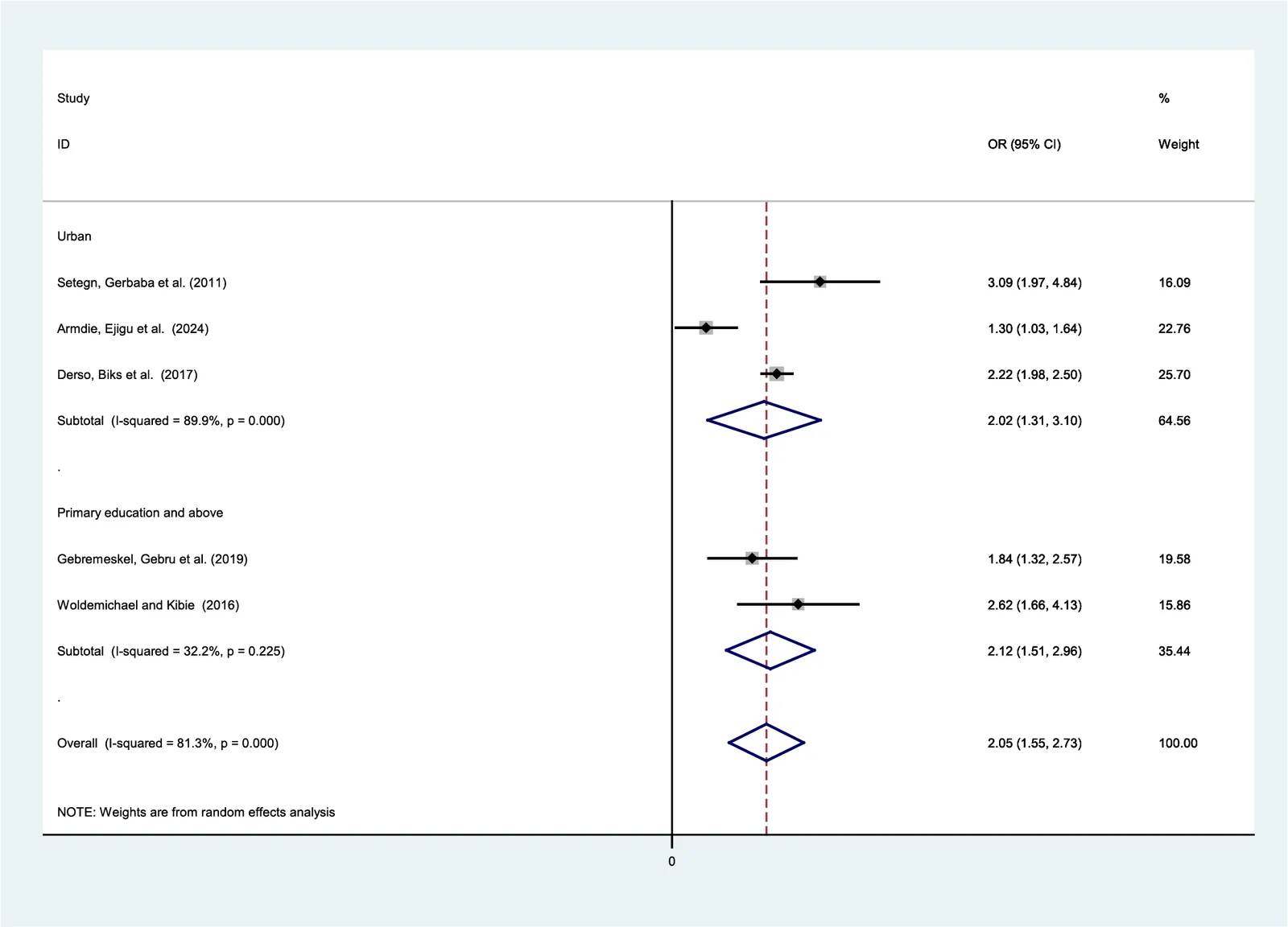
The pooled odds ratio and sociodemographic characteristics of studies included in the review of determinants of EIBF in Ethiopia

Our findings showed that residence, counselling during antenatal care, postnatal care advice, spontaneous vertex delivery (SVD), health institution and pre-lacteal feeding variables were among the determinants that indicated a significant association with EIBF in Ethiopia. The pooled odds ratio from three studies that reported the residence variable: the odds of mothers residing in urban settings were 2 times more likely to initiate breastfeeding than those living in rural settings, with an odds ratio of 2.02 (95% CI: 1.31–3.10) (p-value < 0.001) [18, 29, 44] (Fig. 2).

**Fig. 3:**
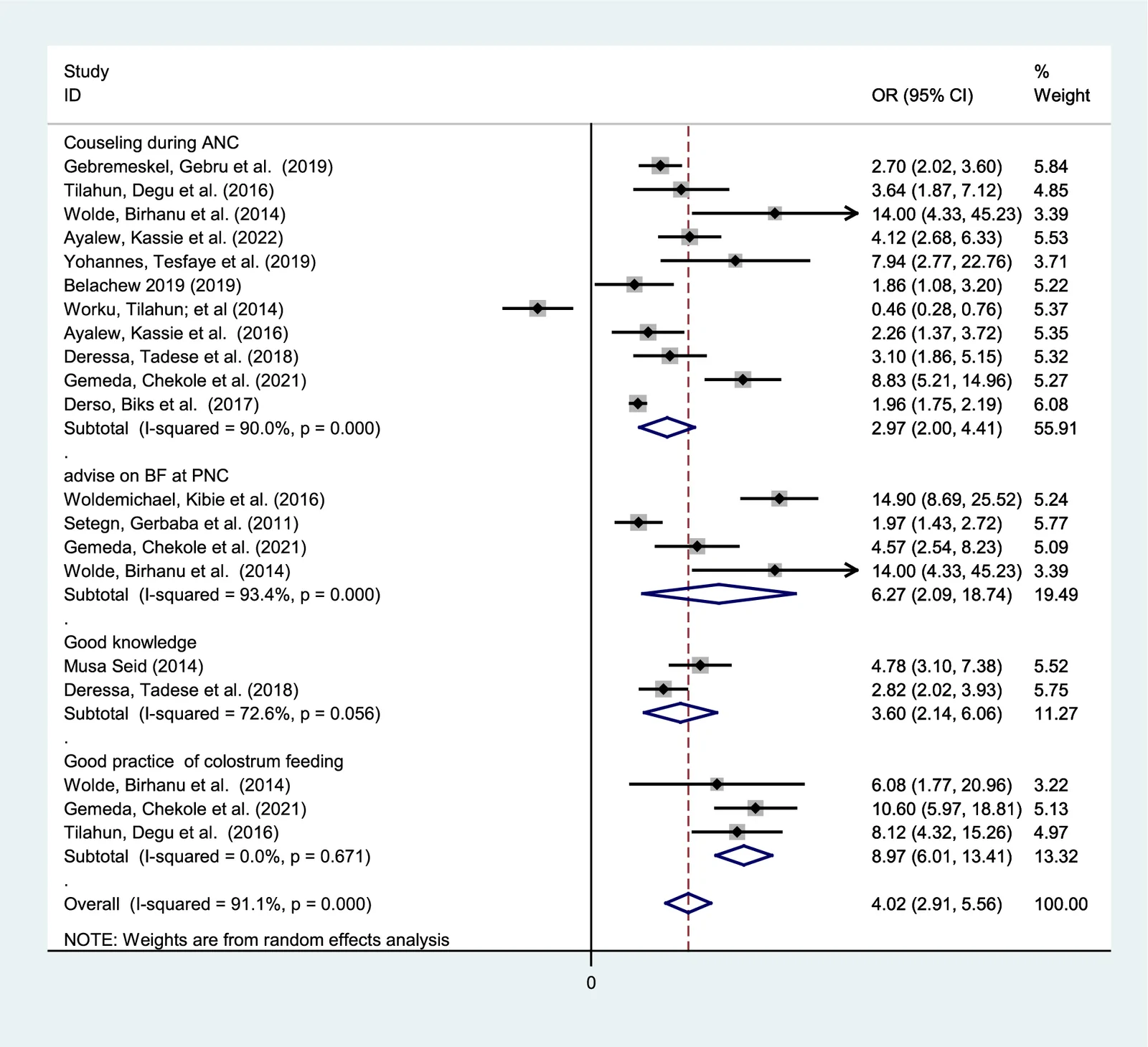
The pooled odds ratio of health- Literacy characteristics of studies included in the review of determinants of EIBF in Ethiopia

In the analysis of the association between counselling during antenatal care and early initiation of breastfeeding in Ethiopia, we considered eleven articles in this variable. Hence, mothers who got counselling during antenatal care were 2.97 times more likely to initiate breastfeeding than their counterparts, with an odds ratio of 2.97 (95% CI: 2.00–4.41) (p-value < 0.001) [29, 39] (Fig. 3).

The pooled estimate of four studies showed that those mothers who received advice during postnatal care were 6.27 times more likely to initiate BF than those who didn’t receive advice during postnatal care, with an odds ratio of 6.27 (95% CI: 2.09–18.74) (P-value < 0.001) [18, 28, 35, 39] (Fig. 3).

**Fig. 4:**
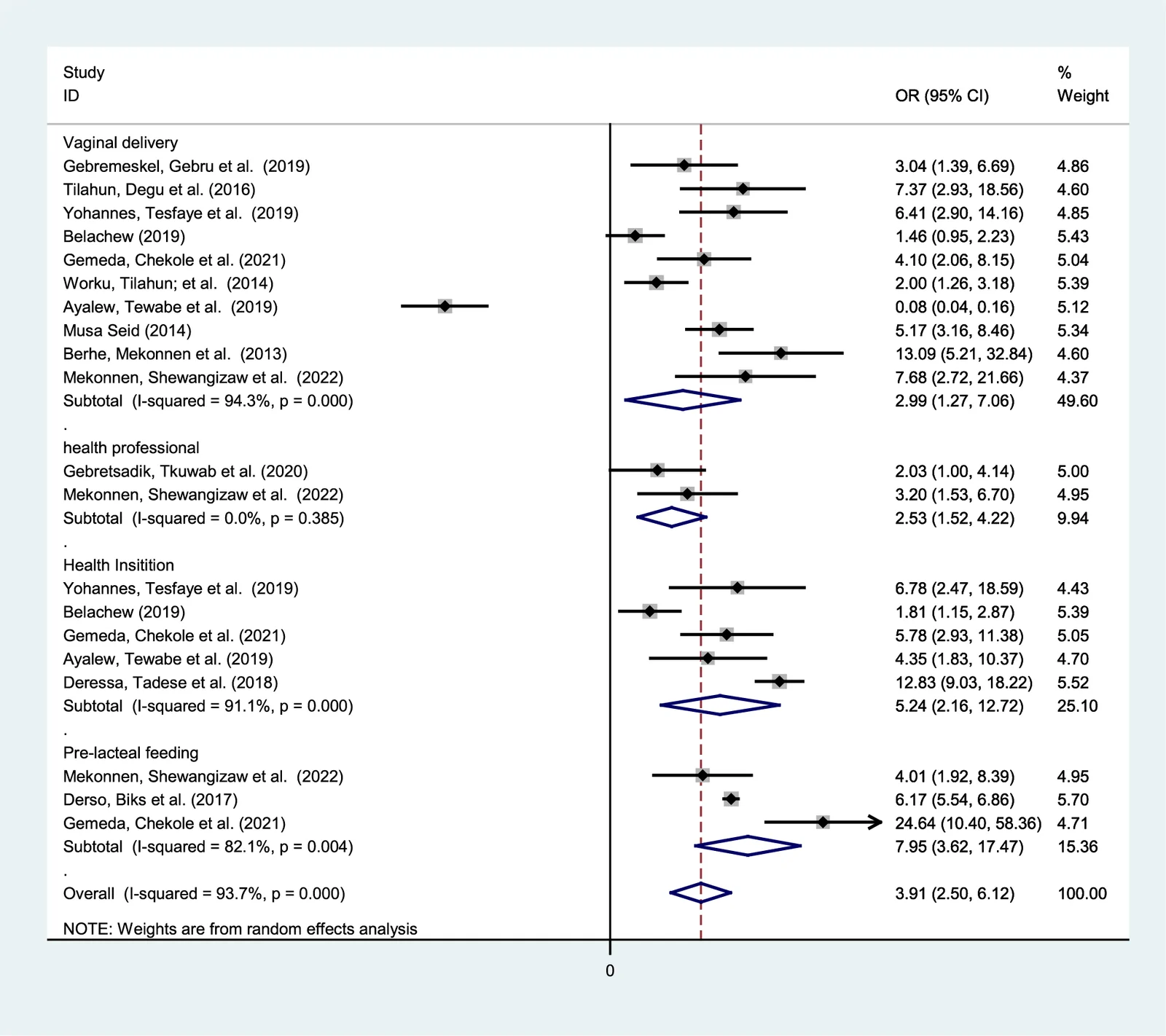
The pooled odds ratio of health-related characteristics of studies included in the review of determinants of EIBF in Ethiopia

The pooled odds ratio of ten studies reported that mothers who had normal delivery (vaginal) were 2.99 times more likely to initiate breastfeeding as compared to those who delivered via caesarean section, with an odds ratio of 2.99 (95% CI: 1.27–7.06) (P-value < 0.001) [31, 32, 34, 38, 40, 42] (Fig. 4).

Five studies were assessed for the association between health institutions and the early initiation of breastfeeding in Ethiopia. Accordingly, we found that mothers who gave birth at health institutions were 5.24 times more likely to initiate breastfeeding than mothers who didn’t give birth at health institutions (OR = 5.24, 95% CI: 2.16–12.74, p-value < 0.001) [31, 33, 35, 38] (Fig. 4).

Moreover, the pooled results of three studies revealed that mothers who did not practice pre-lacteal feeding to their newborns were 7.95 times more likely to initiate breastfeeding early when compared to those who practiced pre-lacteal feeding to their newborns (OR = 7.95, 95% CI: 3.62–17.47, p-value < 0.001) [29, 35, 42] (Fig. 4).

### Publication bias

**Fig. 5:**
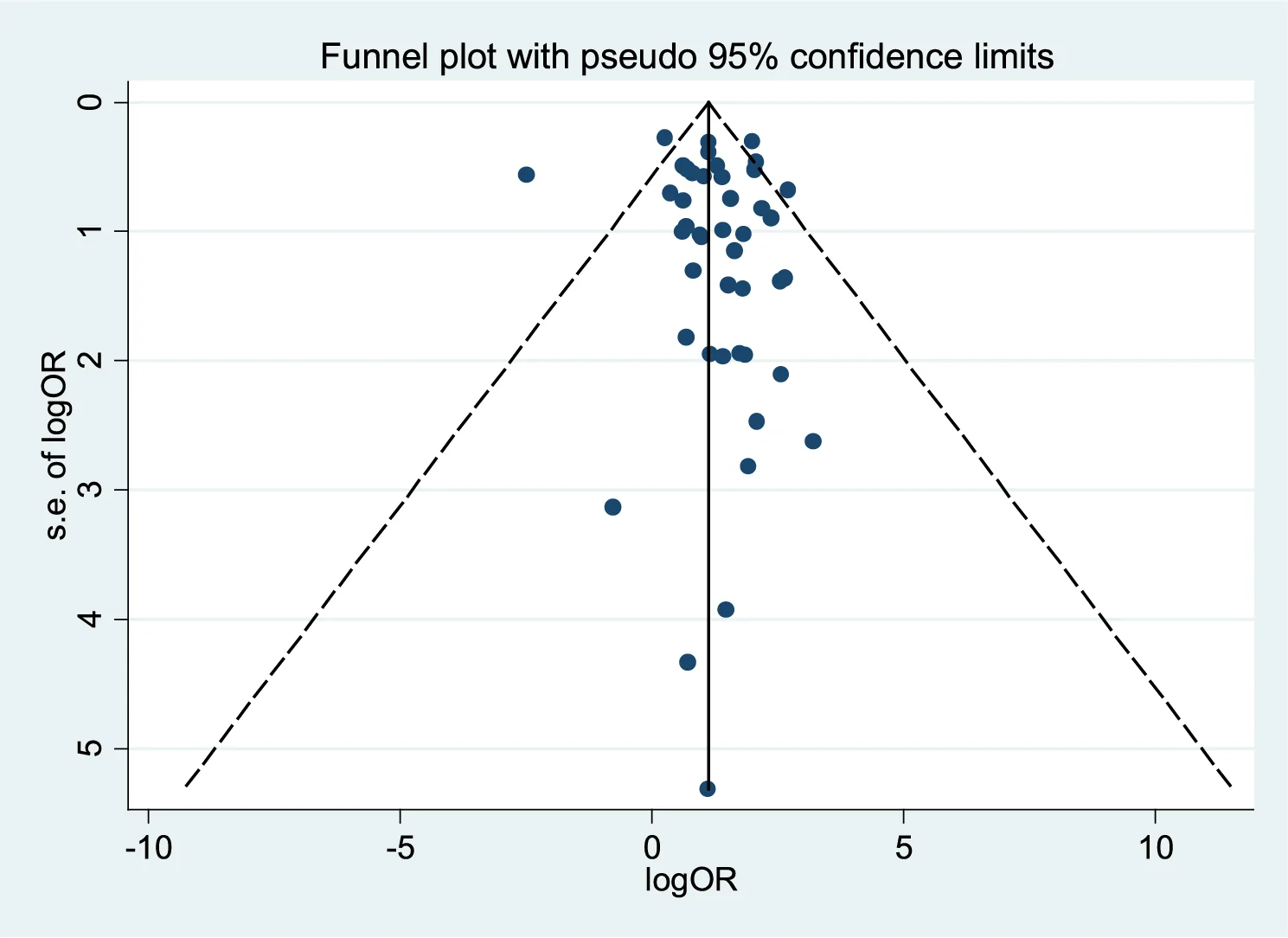
Funnel plot showing the symmetric distribution of articles on EIBF determinant in Ethiopia

Because multiple predictors were evaluated separately, publication bias was checked via Egger’s test for eligible strata containing sufficient primary inputs. For the antenatal counseling stratum (get 10 studies), the visual funnel plot demonstrated acceptable symmetry, and Egger’s regression test confirmed the absence of significant publication bias (*P* = 0.317) (Fig. 5). For strata with fewer than 10 studies (such as residence and pre-lacteal feeding), formal publication bias testing was omitted per epidemiological guidelines to avoid false security. To address the quality of our findings, a formal GRADE certainty assessment was conducted. Due to the high statistical heterogeneity (I^2^ > 50\%) across the primary observational studies, the certainty of evidence for urban residence, ANC counseling, institutional delivery, and mode of delivery was graded as Low. The certainty of evidence for non-practice of pre-lacteal feeding was graded as Moderate due to lower regional variance.

## Discussions

This study investigated the variables associated with early initiation of breastfeeding in Ethiopia through a systematic review and meta-analysis. Based on our review, we identified a range of maternal factors to be significantly associated with EIBF that included residence, counselling during antenatal care, postnatal care advice, SVD, women who gave birth at health institutions and pre-lacteal feeding variables in Ethiopia. According to the pooled odds ratio of three studies included in this meta-analysis, urban residency was reported to be associated with EIBF and concordant with studies done in Vietnam and the multilevel study in Ethiopia that reported urban residency to be more likely to initiate breastfeeding early compared to those who live rural [45, 46]. The possible explanation for this might be the higher percentage of rural women who give birth outside of health facilities and without being attended by a health professional at birth, causing women to miss counselling on early breastfeeding.

Our findings revealed a significant association between counselling during antenatal care and EIBF in Ethiopia. Similar findings were reported by Pakistan and Indian studies [47, 48], which found that receiving antenatal counselling helps mothers initiate early breastfeeding. This could be because EIBF during ANC follow-ups exposes mothers to the benefits of early breastfeeding for both the mother and the child, encouraging them to plan ahead and start breastfeeding on time for their newborn babies [49]. This implies that counselling provided during ANC services to mothers has a higher beneficial effect on EBIF.

The current review also found that women who received advice during postnatal care were more likely to EBIF than those who didn’t receive advice during postnatal care. This supports other previous findings that emphasize that postnatal period follow-up is an opportunity and allows for a more precise assessment of advice during this specific time, potentially accounting for the likelihood of starting early breastfeeding [50, 51].

The current study found a significant association between the mode of delivery and the initiation of breastfeeding. Mothers who had SVD were more likely to initiate breastfeeding early than those who delivered via caesarean section, and several previous studies support our findings. For example, a systematic review and meta-analysis of world literature on breastfeeding after caesarean delivery reported that caesarean delivery was associated with a delay in the initiation of breastfeeding and lower breastfeeding rates at discharge and at six weeks postpartum [52]. Another study in the same fashion documented caesarean delivery as a significant barrier to the initiation of early breastfeeding due to delayed mother-infant bonding and the physical recovery required after surgery [53]. Biologically, the physiological stress of surgery, combined with delayed post-operative skin-to-skin contact and anesthesia-induced infant lethargy, delays maternal oxytocin surge and prolactin secretion, inhibiting immediate lactation pathways. In contrast, mothers who delivered babies by caesarean were equally likely to start breastfeeding after receiving sufficient postnatal care as mothers who delivered babies vaginally [54], indicating that the mode of delivery has less effect on EIBF.

In this study, mothers who gave birth at health facilities (HF) were more likely to initiate breastfeeding than their referent groups. This assertion was confirmed by the study done in Ghana, in which they stated that mothers who delivered in health facilities were more likely to initiate breastfeeding within the first hour of birth compared to those who delivered at home [55]. This is likely since health facilities often attend deliveries and provide counselling as deemed necessary, as shown in the Nepal study [56]. In addition, most HFs often have protocols and practices that promote immediate skin-to-skin contact and breastfeeding initiation [57]. This aligns with the principle that HF arranges for a conducive environment for breastfeeding education and support, which may explain the amplified likelihood observed in the current study.

In contrast to our findings, other researchers highlight that the influence of the delivery setting on the commencement of breastfeeding can differ depending on the cultural and regional context as well as the calibre of care given. Regardless of the place of birth, breastfeeding habits can be greatly influenced by customs and beliefs in some areas. Initiating breastfeeding was highly impacted by traditional postpartum practices and family support systems, sometimes to a greater extent than institutional birth, according to a study conducted in West Africa [58], and sometimes due to poor healthcare quality and income status [59].

In line with our findings, several studies have demonstrated that the absence of pre-lacteal feeding is significantly associated with EIBF [18, 60]. Contrary to our findings, the Kenyan and Indian studies reported that pre-lacteal feeding is inconclusive [61, 62], demonstrating that other socio-cultural and healthcare factors are involved in EBIF.

### Strength and limitations

Inclusion of the studies that assessed EBIF contextually and extensive searches using different searching strategies (manual and electronic) were the strengths of this review. Data extraction was also conducted using a predetermined tool and was extracted by two authors independently to minimize bias. Quality assessment was also done by two independent authors. There are several limitations to this study. First, we did not do subgroup analyses by region because there weren’t enough studies that must be explicitly recognized when interpreting our pooled figures. First, because our final data structure consists entirely of cross-sectional observational studies, we cannot infer definitive causal links between any identified predictor and EIBF; our models only indicate strong associative indicators. Second, we noted high statistical heterogeneity ($I^2 > 50\%$) across almost all major sub-analyses. While we utilized a random-effects model to buffer these variances, residual confounding from localized cultural habits, varying definitions of rural parameters, or maternal age structures across primary datasets cannot be completely ruled out. Third, due to the limited number of high-quality primary papers containing fully adjusted multi-variable datasets in specific regions of Ethiopia, we were unable to conduct a robust regional subgroup analysis. Finally, our decision to exclude grey literature to protect data quality control might have inadvertently omitted localized institutional reports containing unique regional findings. These factors collectively led to a ‘Low’ certainty rating for several pooled predictors within our GRADE evaluation.

### Conclusion & implications

This systematic review and meta-analysis consolidate the critical structural, institutional, and behavioral predictors driving the early initiation of breastfeeding (EIBF) in Ethiopia. The findings demonstrate that maternal residence, antenatal and postnatal counseling, institutional delivery, vaginal birth, and the avoidance of pre-lacteal feeds are powerful prognostic indicators of timely breastfeeding practices.

To effectively translate these empirical insights into actionable health strategies, a coordinated, multi-tier approach is required across public health policy, clinical practice, and academic research as described subsequently;

Firstly, Policies should focus on subsidizing and institutionalizing comprehensive prenatal and postnatal counseling packages that specifically reach underserved agrarian populations to bridge the urban-rural gap.

Secondly, Midwifery and obstetric protocols within Ethiopian health centers must be redesigned to accommodate immediate skin-to-skin contact for mothers undergoing cesarean deliveries, ensuring that post-operative physical barriers do not disrupt early infant bonding. Furthermore, clinical staff must strictly enforce facility-level bans on non-medically indicated pre-lacteal feeding.

Thirdly, there is a need for large-scale, prospective cohort studies across diverse regional states in Ethiopia to track long-term maternal feeding behaviors and clarify the exact causal pathways linking clinical delivery environments to neonatal feeding outcomes.

## Supplementary Information

Supplementary Material 1.

## Data Availability

No datasets were generated or analysed during the current study.

## Declarations

### Annexes

STATA Do file for determinants.

## Abbreviations

AJOL: African Journal Online
ANC: Antenatal care
AOR: Adjusted odds ratio
BF: Breastfeeding
DHS: Demographic and Health Surveys
EIBF: Early initiation of breast feeding
HF: Health facilities (HF)
JBI-MAStARI: Joanna Briggs Institute Meta-Analysis of Statistics Assessment and Review Instrument
MeSH: Medical Subject Headings
OR: Odds ratio
PNC: Postnatal care
PRISMA: Preferred Reporting Items for Systematic Reviews and Meta-Analyses
SVD: Spontaneous vertex delivery
WHO: World Health Organization; WSO: World Stroke Organization
